# Pathological features of Breast Cancer seen in Northwestern Tanzania: a nine years retrospective study

**DOI:** 10.1186/1756-0500-4-214

**Published:** 2011-06-22

**Authors:** Peter F Rambau, Philipo L Chalya, Mange M Manyama, Kahima J Jackson

**Affiliations:** 1 Department of Pathology Weill Bugando University College of Health Sciences, Box 1464 Mwanza, (Bugando Street) Postal code +255, Tanzania; 2 Department of Surgery Weill Bugando University College of Health Sciences, Box 1464 Mwanza, (Bugando Street) Postal code +255, Tanzania; 3 Department of Anatomy Weill Bugando University College of Health Sciences, Box 1464 Mwanza, (Bugando Street) Postal code +255, Tanzania; 4 Department of Pathology Bugando Medical Center, Box 1370 Mwanza, (Bugando Street) Postal code +255, Tanzania

**Keywords:** Breast Cancer, North Western Tanzania

## Abstract

**Background:**

Breast cancer is more common in Western Countries compared to African populations. However in African population, it appears that the disease tends to be more aggressive and occurring at a relatively young age at the time of presentation. The aim of this study was to describe the trend of Breast Cancer in Northwestern Tanzania.

**Methods:**

This was a retrospective study which involved all cases of breast cancer diagnosed histologically at Bugando Medical Center from 2002 to 2010. Histological results and slides were retrieved from the records in the Pathology department, clinical information and demographic data for patients were retrieved from surgical wards and department of medical records. Histology slides were re-evaluated for the histological type, grade (By modified Bloom-Richardson score), and presence of necrosis and skin involvement. Data was entered and analyzed by SPSS computer software version 15.

**Findings:**

There were 328 patients histologically confirmed to have breast cancer, the mean age at diagnosis was 48.7 years (+/- 13.1). About half of the patients (52.4%) were below 46 years of age, and this group of patients had significantly higher tendency for lymph node metastasis (p = 0.012). The tumor size ranged from 1 cm to 18 cm in diameter with average (mean) of 5.5 cm (+/- 2.5), and median size of 6 cm. Size of the tumor (above 6 cm in diameter) and presence of necrosis within the tumor was significantly associated with high rate of lymph node metastasis (p = 0.000). Of all patients, 64% were at clinical stage III (specifically IIIB) and 70.4% had lymph node metastasis at the time of diagnosis. Only 4.3% of the patients were in clinical stage I at the time of diagnosis. Majority of the patients had invasive ductal carcinoma (91.5%) followed by mucinous carcinoma (5.2%), Invasive lobular carcinoma (3%) and in situ ductal carcinoma (0.3%). In all patients, 185 (56.4%) had tumor with histological grade 3.

**Conclusion:**

Breast cancer in this region show a trend towards relative young age at diagnosis with advanced stage at diagnosis and high rate of lymph node metastasis. Poor Referral system, lack of screening programs and natural aggressive biological behavior of tumor may contribute to advanced disease at the time of diagnosis.

## Background

Breast cancer is a significant cause of mortality worldwide, with a prevalence that is estimated to be 1,000,000 annually[1]. In African women, the diagnosis is often made between 35 and 45 years of age. This is fifteen years earlier than women in Europe and North America. One study showed the mean age of diagnosis in black patient was 57.6 years with large tumor size compared to 62.6 years in white patients. The overall incidence was lower in black women although for patients younger than 40 years the incidence was higher by 20% in black women[2]. The estimated age standardized rates for breast cancer incidence in sub-Saharan Africa range from 15 to 53 per 100, 000 women, which is lower than what is seen in Western countries[3]. The mortality rate tends to be high among women in sub-Saharan Africa as tumors tend to be very aggressive with short periods of time between the onset of symptoms and diagnosis[4].

In Tanzania, breast cancer accounted for 8.1% of all female cancers in years 1974-1987, and most of the patients were under 30 years of age[5]. A study done in Dar-es-Salaam on 50 patients showed that most patients presented with advanced stage with no single patient in stage I, and the the majority were in stage IIIB[6]. In other parts of the world older women are at high risk, where approximately 77% of the breast cancer occurs in women over 50 years[7], but this trend is somehow different in Africans where the disease is common at young age[3]. In sub Saharan Africa, the disease is seen commonly in women below 30 years of age[8].

Few studies have shown that breast cancer in Africans tends to present at a young age with advanced aggressive disease with a poor prognosis compared to Western white females[4,9,10]. Furthermore, breast cancer in Africa shows the trend of rapid increase[11].

Several factors have been implicated in the prognosis of the patients with breast cancer. Age has been shown to be an important factor in the prognosis of breast cancer, where by disease presentation at young age has shown a worse prognosis than in older age[12]. This has been explained by the fact that cancer at young age tends to be more aggressive and biological behavior could be different. In Africans, breast cancer tends to present at young age and this could be a reasons for bad prognosis in these patients [4,8-10].

The clinical stage of the disease at presentation is important for the outcome of the patient with breast cancer. In most African patients, especially at young age, the disease present at an advanced stage [3,8]. This can be explained by delay in seeking medical services, poor medical service with no screening programs for breast cancer. The staging of the patient in conjunction with histological grade can be used to determine the prognosis of the patient. Whereas stage I disease has about 96% 5-year survival, this can be as low as 18% 5-year survival rate in stage IV. By using histological grade alone, histological grade I has about 93% 5-year survival. Stage I disease with histological grade I has shown a 99% 5-year survival compared to only 13% 5-year survival in stage IV with histological grade III[13-15]. Histological grade used is that described by Bloom & Richardson[16].

One less common histological type of breast cancer called centrally necrotizing carcinoma of the breast has shown to be more aggressive by having early metastasis and rapid clinical progression and it was found to be good for determining prognosis in pre-menopausal women [17,18]. This study was therefore, carried to describe clinical and pathological features of patients with breast cancer in this region and compare the trends with other regions.

## Methods

This was a retrospective study of histologically confirmed cases of breast cancer seen at the department of Pathology of Bugando Medical Centre (BMC) over a period of 9-years between January 2002 and September 2010. BMC is a consultant, tertiary care and teaching hospital for the Weill-Bugando University Collage of Health Sciences (WBHCHS) with a bed capacity of 870. Subjects of this study included all histologically confirmed breast cancer made during the period of 2002 to 2010. Data was retrieved from the records in the Pathology department. Histology slides were re-evaluated for histological type; and grade was established by Modified Bloom-Richardson score system which scores for tubular formation, nuclear pleomorphism and mitotic rate within tumor cells. Other tumor morphological features like presence of necrosis, and involvement of the skin was evaluated. The clinical stage of the disease was assigned to each patient by using TNM (AJCC cancer staging manual); this is a staging system which is expression of anatomical extent of disease based on extent of primary tumor (T), absence or presence of and extent of regional lymph node metastasis (N) and absence or presence of distant metastasis. The clinical information and demographic information was obtained from Patients' files kept in the Medical record department and the surgical wards. Patients with incomplete data were excluded from the study. Ethical approval to conduct the study was obtained from the WBUCHS/BMC joint institutional ethic review committee before the commencement of the study. Data were entered and analyzed using SPSS computer software version 15. Being a retrospective study, it was not possible to establish exact time period of the onset of the disease, and follow up of the patients was not possible and survival could not be established. By doing prospective study the patients can be properly followed and patient's survival and prognostic factors can be established.

## Results

In this study there were 328 patients with histologically confirmed breast cancer, the mean age at diagnosis was 47.8 (+/- 13) and the median was 46 years. 30.2% of the patients were in age group of 30 - 40 years, followed by 41-50 years (25.9%), and age group below 30 years were 21% and only 17.4% were above 60 years (Table 1). The tumor size ranged from 1 cm to 18 cm in diameter with average (mean) of 5.5 cm (+/- 2.5). Majority of the patients (64%) were at clinical stage III, and 70.4% of the patients had lymph node metastasis at the time of diagnosis (Table 1)

**Table 1:** Patients age profile, clinical stage and lymph Node metastasis at the time of diagnosis

Age group (years)	N (%)
Below 30	21(6.4)
30-40	99(30.2)
41-50	85(25.9)
51-60	66(20.1)
Above 60	57(17.4)
**Stage at the time of diagnosis**	
O	1 (0.3)
I	14 (4.3)
II	68 (20.7)
III	210(64.0)
IV	35 (10.7)
**Lymph node metastasis**	
Positive	231 (70.4)
Negative	97 (29.6)

Total	328

**Figure 1:**
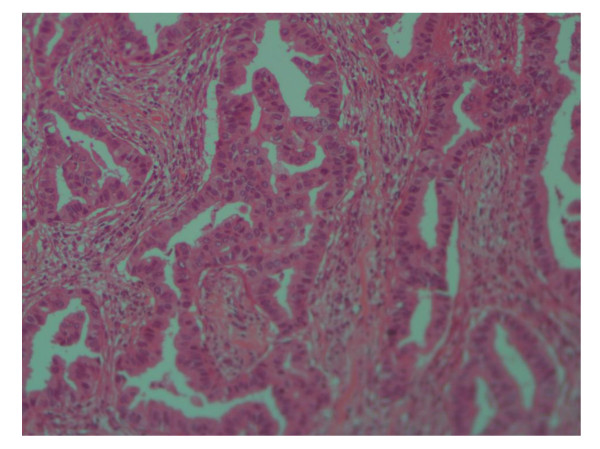
**Photomicrograph of Invasive ductal carcinoma of the breast grade 2, H and E stain (×10)**.

**Figure 2:**
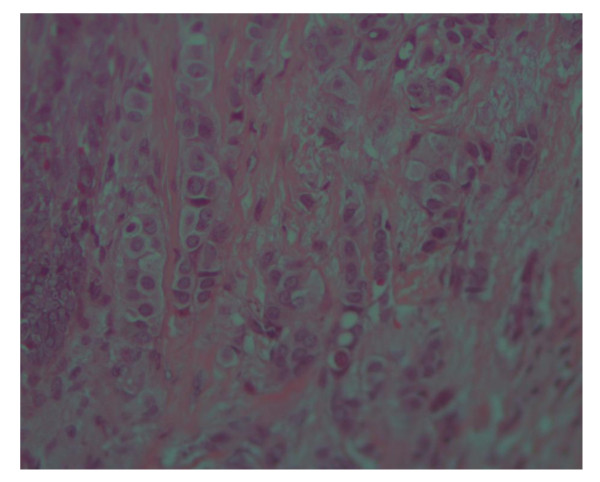
**Photomicrograph of Invasive lobular carcinoma of the breast with Typical Indian file pattern of Infiltration, H and E stain (×20)**.

**Figure 3:**
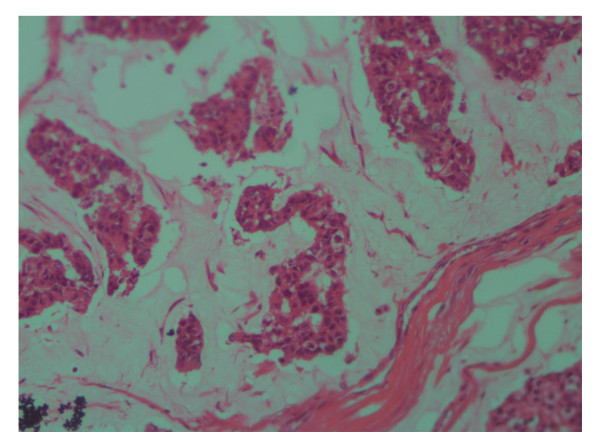
**Photomicrograph of mucinous carcinoma of the breast, a cluster of tumor cells in a pool of mucin, H and E stain (×20)**.

Of all patients, 91.5% (300) had invasive ductal carcinoma, 3% (10) has Invasive lobular carcinoma and 5.2% (17) had mucinous carcinoma (Figure 1), [Figure 2] and [Figure 3], only one patient had in situ ductal carcinoma. 56.4% (185) of the patients had histological grade 3, 42.1%(138) grade 2 and 1.5% (5) grade 1.

Tumor size was significantly associated with rate of lymph node metastasis. Patients with tumors above 6 cm which was the median size, had high rate of lymph node metastasis compared to patients with tumor below 6 cm [p = 0.000] (Table 2).

**Table 2:** Tumor size in association with lymph node metastasis

	Lymph node positive N (%)	Total	p-value
**Tumor size**			
			
**Below 6 cm**	100 (57.8)	173	
**Above 6 cm**	131 (84.5)	155	0.000

**Total**	**231**	**328**	

Patients below 46 years of age (median age) also had high rate of lymph node metastasis compared to the patients above median age (p = 0.012) (Table 3). Presence of necrosis within the tumor was significantly found in tumors above 6 cm [p = 0.000] (Table 4) and such tumors were commonly seen in patients below 46 years of age [p = 0.012] (Table 5), however the presence of necrosis within the tumor was not associated with rate of lymph node metastasis (p = 0.205).

**Table 3:** Patient age in association with Lymph node metastasis

Age group (years)	Lymph node positive N (%)	Total	p-value
**Less than/equal 46**	130 (75.5)	172	
**Above46**	101 (64.7)	156	0.012

**Total**	231	328	

**Table 4:** Tumor size in association with necrosis

Tumor size (cm)	Presence of necrosis N (%)	Total	p-value
Below 6	59 (34)	173	
Above 6	86 (55.4)	155	0.000

Total	145	328	

**Table 5:** Tumor size in association with patient's age

	Age less/equal 45 yrs	Age above 45 yrs	Total	p-value
Tumor size (cm)				
				
Below 6	80	93	173	
Above 6	92	63	155	0.012

**Total**	172	156	328	

Tumors with low histological grade had low rate of lymph node metastasis compared to high grade tumors, statistical association was not established because only 5 patients (1.6%) had low grade tumors. All grade one tumors were below 6 cm in diameter, 58% of grade two were below 6 cm, and 47.6% of grade three tumors were below 6 cm, there was a trend of high grade tumors to be of larger size though association could not be established. Ulcerated tumors were commonly seen in grade three tumors, and high grade tumors were also common in patients with less than 46 years of age.

Managements of those patients included mastectomy with axillary lymph node clearance followed by adjuvant hormonal therapy or chemotherapy. A patient with advanced disease, down staging of the tumor was done by radiation followed by surgery. Radiation and chemotherapy was used as palliative care for patients with advanced inoperable disease.

## Discussion

In this study the mean age at diagnosis was 47.8 years, The findings are the same as that seen in Ghana where the mean age at diagnosis was 48 years[19]. Our finding shows that 17.4% of patients were above 60 years of age, which is the mean age of diagnosis in African American (60.8 years) and in white Americans (62.4 years). It has been reported that the occurrence of breast cancer at young age is associated with a worst prognosis and thus prognosis improves with age, with the best prognosis in patients over 75 years [20]. Similar findings were also seen in Nigeria and Senegal where the mean age at diagnosis was 44.8, with majority of tumors being of high grade, associated with advanced age and lymph node involvement [21]. In our study, 70.4% had lymph node involvement at the time of diagnosis while 64% were in stage III (specifically IIB) which is advanced disease. In addition the mean tumor size was larger (5.5 +/- 2.5) compared to that seen in Nigeria and Senegal (4.4 +/- 2.0 cm) and histological high grade tumors were 56.4% which is relatively lower. A study in Uganda which is neighboring country reported the median age at diagnosis to be 45 years with peak age group of 30-39 years[22], This trend is the same as seen in this study. Similarly the study in British black women showed the median age at diagnosis was 46 years as seen in this study, and these patients had also aggressive disease with more of histology grade 3, high rate of lymph node metastasis; and majority had triple negative tumors with basal like features[23]. There is a high possibility that our patients might have a lot of triple negative, this is an area which need further investigation. Many studies showed that as the age increases the tumors tends to be less aggressive with low rate of lymph node metastasis, low clinical stage and high levels of ER and PR expression. In younger patients, the tumors tend to be of unfavorable histological features with low receptor status [24]. Another study showed the age of 80 years and above to have more favorable histological features[25], this findings are also seen in our study where patients below 46 years of age had high rate of lymph node metastasis (p = 0.012)

In this study the tumor size was significantly associated with presence of necrosis within the tumor and rate of lymph node metastasis. Also large tumors were seen to be of high histological grade although association could not be established due to few numbers of patients in grade one. In this study, large tumor size was associated with younger age (below 46 years), and this could explain the reasons for aggressive disease at younger age. One study showed tumor with necrosis and ER-/PR-were good predictors of metastasis in the lungs[26], and observation from this study showed majority of patients who had metastasis presents with chest symptoms, and with this age profile and histological features there is like hood that majority of our patients are ER-/PR-. Similarly a study in Nigeria showed statistically significant difference in the extent of necrosis in the tumors of postmenopausal and premenopausal patients. Lymph node-positive tumors had more necrosis than lymph node-negative tumors[27]. In this study Necrosis was not associated with lymph node metastasis. Some studies had demonstrated that necrosis is linked to poor prognosis; and necrosis was a feature of tumors possessing an aggressive phenotype (high tumor grade), large size tumor and low estrogen receptor status[28]. In this study necrosis was seen frequently in high grade tumors than low grade tumors, however due to few patients with grade I the statistical difference could not be validated.

Ductal carcinoma was the commonest histological type, which is similar to what is known in literature [29]. In this study 5.2% (17) were mucinous tumors, this was the second to infiltrating ductal carcinoma, and 10 patients out of 17 were above 46 years of age. The median age of patients with mucinous tumors was 55 years, this is relatively younger compared to other studies, however one study showed this tumors to occur at mean age of 55 years, while another study showed mean age was 53 [30,31]. Both studies showed that Mucinous tumor had tendency to have better prognosis, low recurrence rate and low rate of lymph node metastasis. Lymph node metastasis in mucinous tumor is commonly associated with large tumors as seen in one study (means size of 2.7 cm) [32], the high rate of lymph node metastasis seen in our patients could be accounted for by the large tumor size at the time of diagnosis.

## Conclusion

This study showed relatively young age profile of patients with breast cancer in this region, with high rate of lymph node metastasis and advanced clinical stage at the time of diagnosis. This study didn't able to establish exact time onset of the disease and its progress, which needs further investigation. Poor Referral system, natural aggressive biological behavior of tumor may contribute to advanced disease at the time of diagnosis. Raising the public awareness and introduction of screening method can reduce mortality from breast cancer in this community.

## Ethical Approval

The research proposal was presented to joint Bugando Hospital and Weil Bugando University College of Health sciences ethic committee, research and publication where it was approved.

## Consent

This research was retrospective study, histological slides, tissue blocks and patients files were used to get the information, by the time of the research there was no patients available for consent, confidentiality of the patients was maintained, only histology numbers and file numbers were used, this was approved by joint Bugando Hospital and Weil Bugando University College of Health sciences ethic committee, research and publication

## Competing interests

The authors declare that they have no competing interests.

## Authors' contributions

**PR: **Main author of the study, involved in design, writing the proposal, data collection, analysis and preparation of the manuscript. **PC: **Involved in preparation of the study, data collection, clinical staging and preparation of the manuscript. **MM: **Involved in developing the proposal, data collection and preparation of the manuscript. **KJ: **Involved in development of proposal, together with main author in review of histological slides and manuscript preparation. All the authors read and approved the final manuscript.
